# A Questionnaire Study on Patient Perspectives of Sedation for Ophthalmic Procedures

**DOI:** 10.7759/cureus.82385

**Published:** 2025-04-16

**Authors:** Muhammad Awan, Alec J Lippmann, Shayan Samavati, Thomas Dao, William Gannon, Martin Clemmons

**Affiliations:** 1 Clinical Sciences, Alabama College of Osteopathic Medicine, Dothan, USA; 2 Medicine, Alabama College of Osteopathic Medicine, Dothan, USA; 3 Ophthalmology, Eye Surgical Associates, Dothan, USA; 4 Internal Medicine, Alabama College of Osteopathic Medicine, Dothan, USA

**Keywords:** benzodiazepine, eye procedure, eye surgery, ophthalmology, patient opinion, sedation, survey

## Abstract

Introduction

Ophthalmic procedures, such as cataract and refractive surgery, are prevalent in the United States and are often associated with significant preoperative anxiety among patients, which can impact patient comfort and surgical outcomes. Sedation is used in the vast majority of these procedures to reduce anxiety and improve patient cooperation. However, there is limited information on patient-reported experiences and perspectives. This study thus aimed to explore the opinions of patients on sedation for their eye procedures.

Methods

We administered an anonymous Qualtrics survey to gather data such as patient anxiety levels before and after sedation, patient satisfaction, potential side effects from sedatives, and perceived need for sedation in future procedures. The survey was posted in ophthalmology surgery clinics and online.

Results

Analysis of our filtered data found that the preoperative anxiety of patients significantly decreased after receiving sedation, with almost all participants in our survey recommending sedation for ophthalmic procedures to others. Many respondents suggested that resources such as question and answer (Q&A) sessions with ophthalmologists or literature would alleviate concerns about receiving sedation. Although a few mild side effects were self-reported from sedation, a majority of participants did not experience any side effects, and no severe side effects were noted.

Conclusion

These findings suggest that sedation for ophthalmic procedures is successful in reducing patient preoperative anxiety with minimal side effects. However, further research with larger, more diverse populations is warranted to confirm these results and guide future practices in patient care for ophthalmic surgeries.

## Introduction

Ophthalmic procedures, such as cataract extraction, glaucoma treatment, and refractive corrections, are performed daily across the United States (U.S.). While these surgeries are generally safe and effective, the unique nature of ophthalmic interventions, often conducted under topical anesthesia, can lead to considerable preoperative anxiety, as patients remain awake during the procedure. This heightened awareness may result in discomfort and intraoperative movement, impacting surgical outcomes. Between 2012 and 2014, approximately 3.9 million (12.9%) of the 30 million ambulatory procedures in the U.S. were ophthalmic, with lens and cataract surgeries making up 66.5% of these cases [1]. As the aging population grows, the demand for these surgeries is projected to increase significantly. Data from 29 states indicated a 47% rise in ophthalmic surgical workload by 2020, primarily driven by a 67% increase in the elderly population between 2000 and 2050 [1].

Despite advancements in surgical techniques, many patients experience substantial preoperative anxiety, leading to increased physiological stress and potential negative impacts on satisfaction [2]. Sedation plays a crucial role in mitigating these concerns, as it can reduce anxiety, enhance comfort, and promote patient cooperation [2]. Sedation also stabilizes vital signs, which is particularly beneficial for patients with hypertension or other comorbidities [3]. Sedatives are used in up to 90% of ophthalmic procedures and include benzodiazepines, intravenous (IV) anesthetic induction agents, narcotic analgesics, and α-adrenoreceptor agonists [4]. These sedatives typically have a rapid onset and short duration of action, making them suitable for brief procedures like laser-assisted in situ keratomileusis (LASIK) and cataract surgeries. There is no single sedative or regimen that is universally suitable for sedation in eye surgery, but there are indications that drugs such as midazolam, propofol, and remifentanil are the current favorites [5]. The sedative approach in ophthalmic surgery is evolving, with a growing interest in oral sedation as an alternative to IV administration. Oral sedation presents several advantages such as eliminating the need for IV access and reducing costs [6].

Nonetheless, assessing patient satisfaction with these advancements in sedation presents unique challenges, as limited intraoperative communication often leads clinicians to rely on surrogate indicators like patient movement and vital signs. A study by Sadlak et al. found that provider perceptions do not consistently align with patient-reported experiences, highlighting the need for direct patient feedback. Gathering this data is crucial for evaluating sedation effectiveness and optimizing perioperative care [7].

This study aims to explore patient perspectives on sedation during ophthalmic procedures, focusing on their subjective experiences before and after surgery. While previous research primarily addressed the clinical efficacy of sedation, limited data exist on patient-reported satisfaction and emotional responses. Through this investigation, we seek to enhance our understanding of patient experiences, improve counseling, and encourage further research in this important area. Our findings may also provide practical guidance for physicians on discussing sedation options with patients to enhance reassurance and overall satisfaction.

## Materials and methods

In the current study, we aimed to collect insight into patients’ opinions of sedation in ophthalmic procedures, including a comparison of their level of anxiety before and after procedures, their thoughts on sedation, and analysis of potential side effects from sedation. The study was performed through a 16-item Qualtrics survey (https://www.qualtrics.com/en-au/), the content of which was validated by ophthalmologists who agreed to review the survey. Ophthalmologists in Alabama were invited to participate in the project and assist in distributing recruitment flyers to patients. With the permission of the ophthalmologists and their surgery centers, flyers with links to the survey were handed out to all postoperative patients regardless of the eye surgery or procedure performed. The Qualtrics survey was also posted on social media (i.e., Reddit) to connect with patients outside of the ophthalmology surgery centers.

The research protocol was reviewed by the Alabama College of Osteopathic Medicine institutional review board (IRB), and it was approved on the basis that it met the Exempt Category of research involving survey procedures only. Informed consent to participate in the survey was collected on the first page of the Qualtrics survey. The questionnaire was confidential, and no identifying information was collected from the participants. Respondents were not compensated for their participation.

The study population included anyone who had internet access, was age 20 or older at the time of the survey, and had undergone an ophthalmic procedure involving sedation within 12 months of their survey response. These criteria were selected not only to maximize the participant pool but also to reduce the potential recall bias by restricting the recency of the ophthalmic procedure experience. The questionnaire survey was opened in August 2024 for participants to fill out. The survey would close at 50 responses or after six months, whichever came first. A total of 36 responses were received by the end of six months (i.e., February 2025), at which time the survey was closed.

The data was then filtered to exclude responses that were not fully completed or that were completed in under 60 seconds. Responses from participants who were under 20 years of age, did not undergo an eye procedure within 12 months of their response, or did not receive sedation for their procedure were filtered out as well. Microsoft Excel (Microsoft Corporation, Redmond, WA, US) and GraphPad programs (https://www.graphpad.com/) were used to filter and analyze all data and produce graphs for the results.

In the survey, informed consent was first obtained from participants before asking screening questions to determine if the respondents met the inclusion criteria. If the participant did not meet the criteria to continue the questionnaire, the survey would automatically close, and the individual would be thanked for their time. Respondents were then requested to provide demographic information (i.e., birth sex), for which they had the option not to disclose. Participants were asked if they had considered canceling their ophthalmic procedure before receiving sedation out of fear or anxiety. Participants were then asked if they would refuse to undergo future eye procedures if they were told sedation would not be provided.

Respondents were requested to self-report their perceived levels of anxiety regarding the eye procedure before sedation was administered. Participants’ levels of anxiety were measured via a numerical rating scale (NRS) of 0 to 10, where 0 meant no anxiety at all and 10 represented extreme anxiety from the procedure before receiving sedation. Similarly, respondents were asked to self-report their perceived levels of anxiety after sedation was administered. The same NRS scales were used to quantitatively and directly compare the levels of self-reported anxiety before and after sedation.

Subsequently, participants were questioned on how likely they would be to recommend sedation or anti-anxiety medication to family members or friends. The survey concluded by asking respondents about interventions they believed would help them feel more comfortable about receiving sedation (e.g., literature on the topic, support groups), and to self-report any side effects they may have experienced as a result of sedation.

## Results

Survey response rate

Our survey received a total of 36 responses across six months. After filtering data according to the inclusion/exclusion criteria mentioned in the Methods section, as well as removing incomplete responses or those completed in under 60 seconds, 23 survey responses (61.1%) remained.

Participant demographics

Patients were initially asked to self-report their birth sex to better understand patient demographics. Of the respondents, 56.5% (n=13)were female and 43.5% (n=10) were male. No participants self-identified as “Other”, and no participants declined to disclose their birth sex.

Consideration of canceling the procedure before sedation

**Figure 1:**
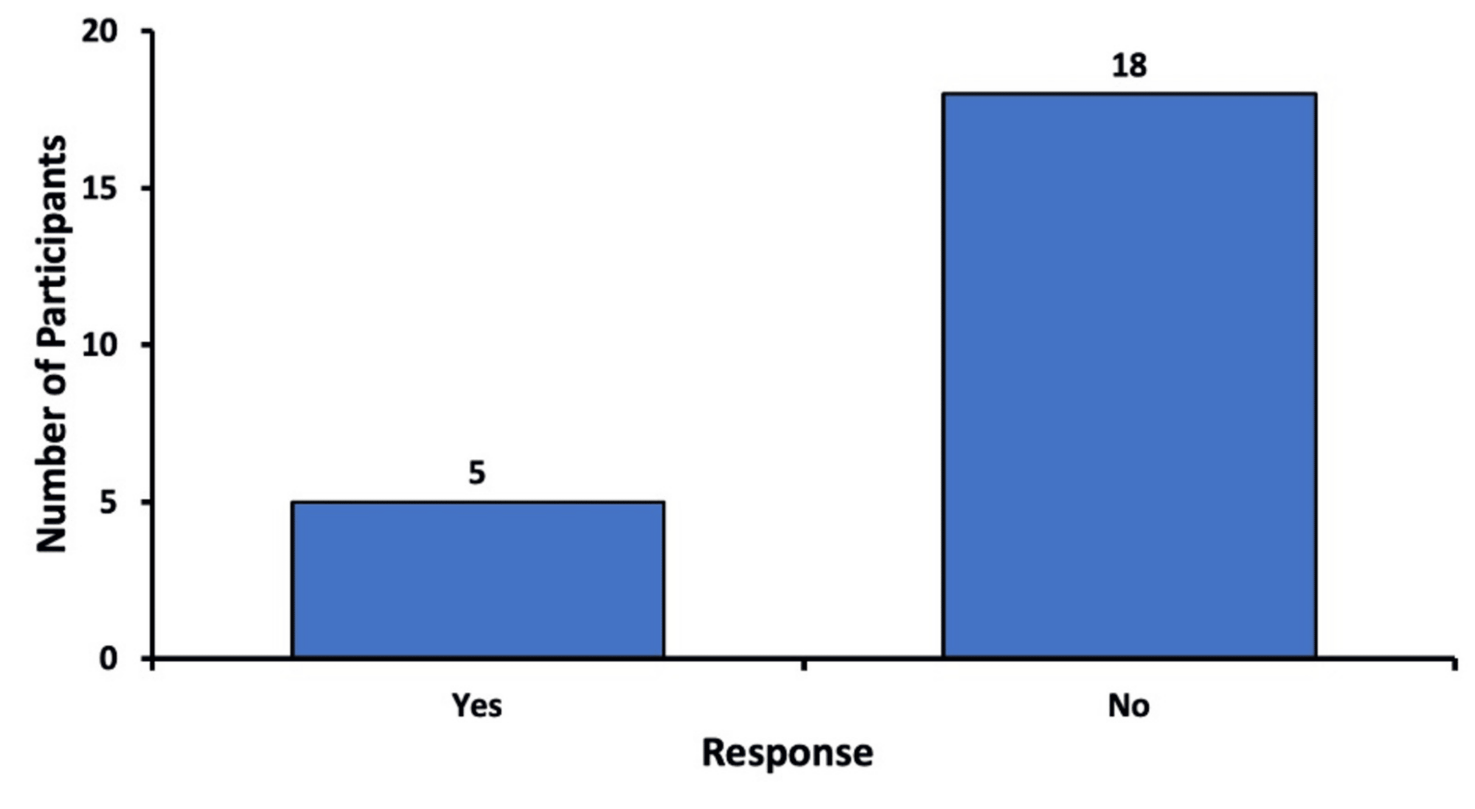
Consideration of canceling the recent procedure before sedation Responses of patients when asked if they had considered a cancellation of their recent eye procedure out of fear, anxiety, or distrust before receiving sedation.

Patients were asked if they had considered canceling or refusing to undergo their recent eye procedure out of fear, anxiety, or distrust before receiving sedation. Of the patients, 21.7% (n=5) reported that they had indeed considered canceling or refusing their procedure before receiving sedation. Of the respondents, 78.3% (n=18) said they did not contemplate cancellation of their procedure or surgery before sedation was administered (Figure 1).

Consideration of canceling future procedures

**Figure 2:**
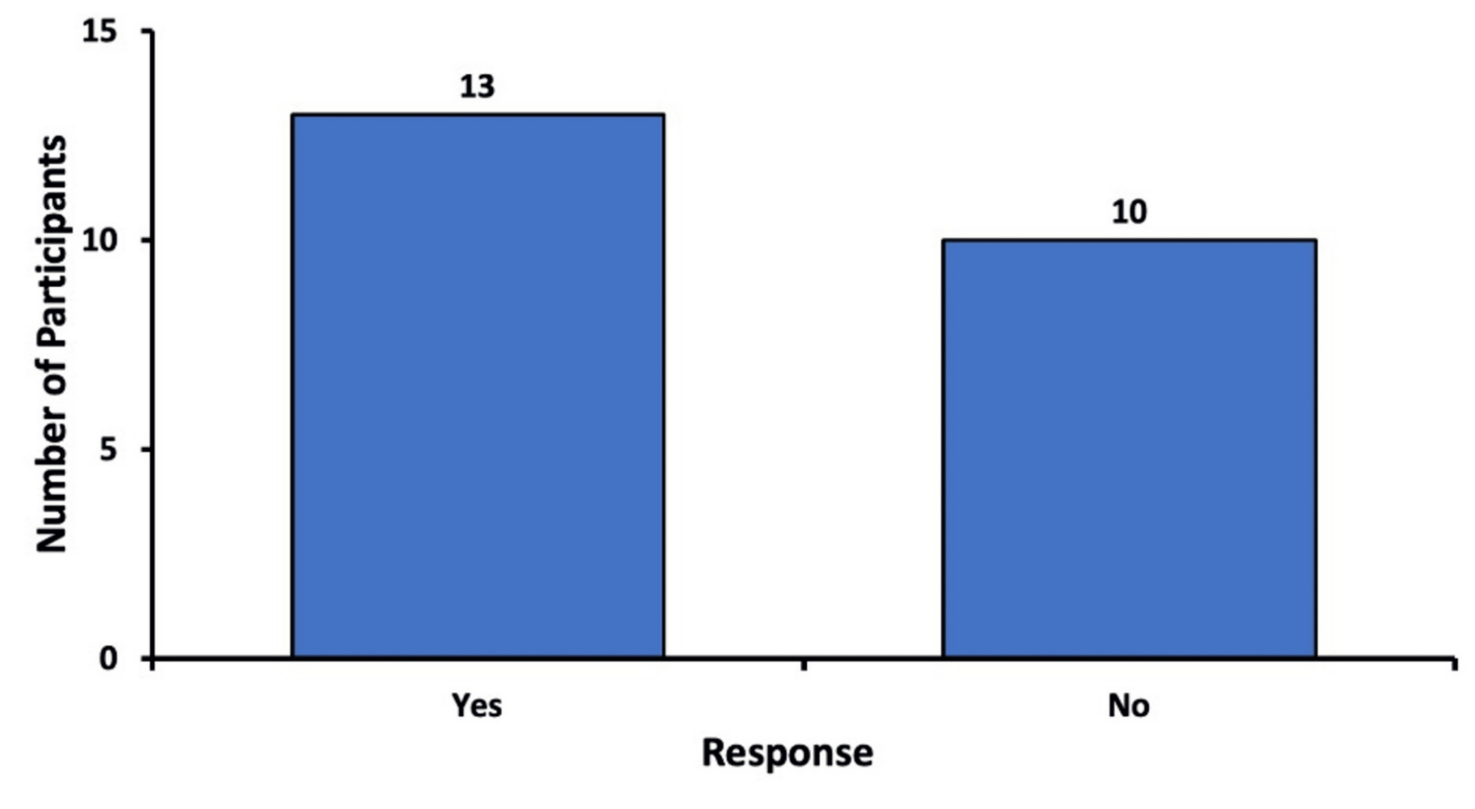
Consideration of canceling future procedures without sedation Responses of patients when asked if they would consider refusing to undergo future eye procedures if they were told that sedation would not be provided

After receiving sedation for their recent procedure, the respondents were asked if they would consider canceling or not undergoing a future eye procedure if they were told that sedatives or anti-anxiety medication would not be administered. Of the participants, 56.5% (n=13) stated that they would indeed consider canceling or refusing to undergo a future procedure or surgery if sedation was not given. Of the respondents, 43.5% (n=10) reported that they would not cancel or refuse a future eye procedure if sedation was not administered (Figure 2).

It must be noted that eight participants who had answered “No” to the question in Figure 1 did respond “Yes” to this question. On the other hand, there were no respondents who answered “Yes” to the question in Figure 1 and then responded “No” to this question.

Comparison of the perceived level of anxiety

**Figure 3:**
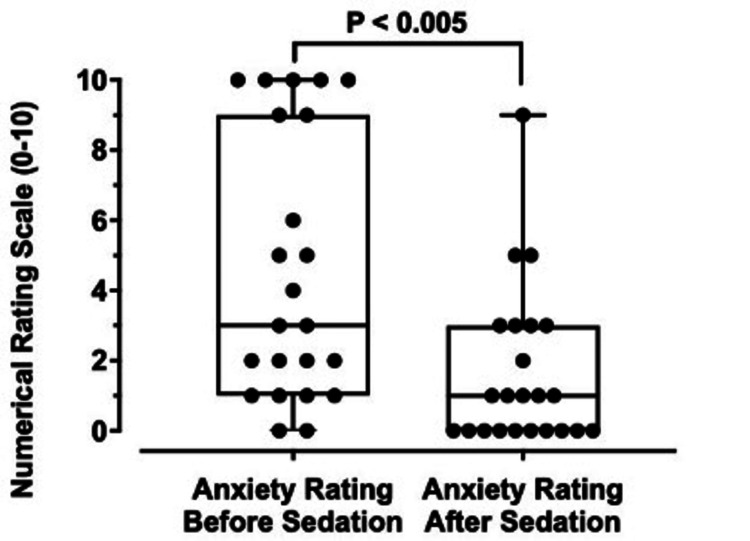
Comparison of anxiety levels before and after sedation Boxplots demonstrating perceived levels of anxiety in participants before and after receiving sedation for their ophthalmic procedures. The average level of anxiety before sedation was received was 4.61 (n=23; range 0 to 10), which decreased to 1.65 (n=23; range 0 to 9) after sedation administration, a reduction of 2.96±0.92 (p < 0.005). The ends of the upper and lower whiskers indicate the maximum and minimum values of each data set, respectively. The interquartile ranges (IQRs) are represented by the open boxes, and the horizontal lines indicate the medians of the data sets.

Participants were asked to self-report their level of perceived anxiety before receiving sedation for their ophthalmic procedure (Figure 3, left). The level of anxiety was measured using a numerical rating scale (NRS), where 0 signified no anxiety at all, and 10 indicated extreme anxiety. The most reported anxiety level before sedation was 10 out of 10 (n=five), with 43.5% (n=10) of respondents reporting an anxiety level of five or above. Two individuals did not have anxiety at all before sedation was administered.

Respondents then provided their level of perceived anxiety after receiving sedation for their eye procedure via the same NRS (Figure 3, right). Ten patients reported complete resolution of their anxiety after sedation (i.e., 0 out of 10 on NRS). The average level of anxiety before the administration of sedation was 4.61, which decreased to 1.65 after receiving sedation, a total reduction of 2.96±0.92 (p-value = 0.0018).

An improvement in anxiety level was seen in 78.3% (n=18) of participants. Eight point seven percent (8.7%; n=2) reported no change in anxiety level after receiving sedation. Of the respondents, 13.0% (n=three) found that their anxiety increased after sedation was administered, one of whom had initially reported an anxiety level of 0 before sedation (Figure 3).

Recommendation of sedation to others

**Figure 4:**
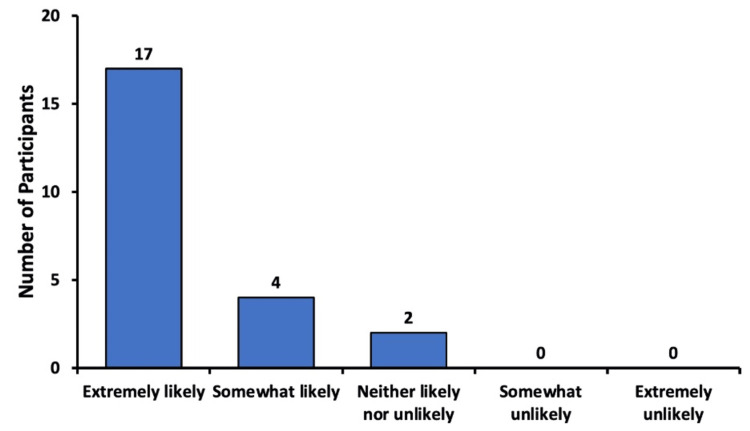
Likelihood of recommending sedation to others Likelihood of participants to recommend sedation for eye procedures to family members or friends

Patients were asked how likely they would be to recommend sedation for eye procedures to a family member or friend. The five possible response options are depicted in Figure 4. Of the respondents, 73.9% (n=17) answered that they were “extremely likely” to recommend sedation to others. Of the participants, 17.4% (n=4) stated that they were “somewhat likely” to recommend sedation, and 8.7% (n=2) answered “neither likely nor unlikely”. No patients responded with “somewhat unlikely” or “extremely unlikely” (Figure 4).

Interventions to improve the sedation experience

**Figure 5:**
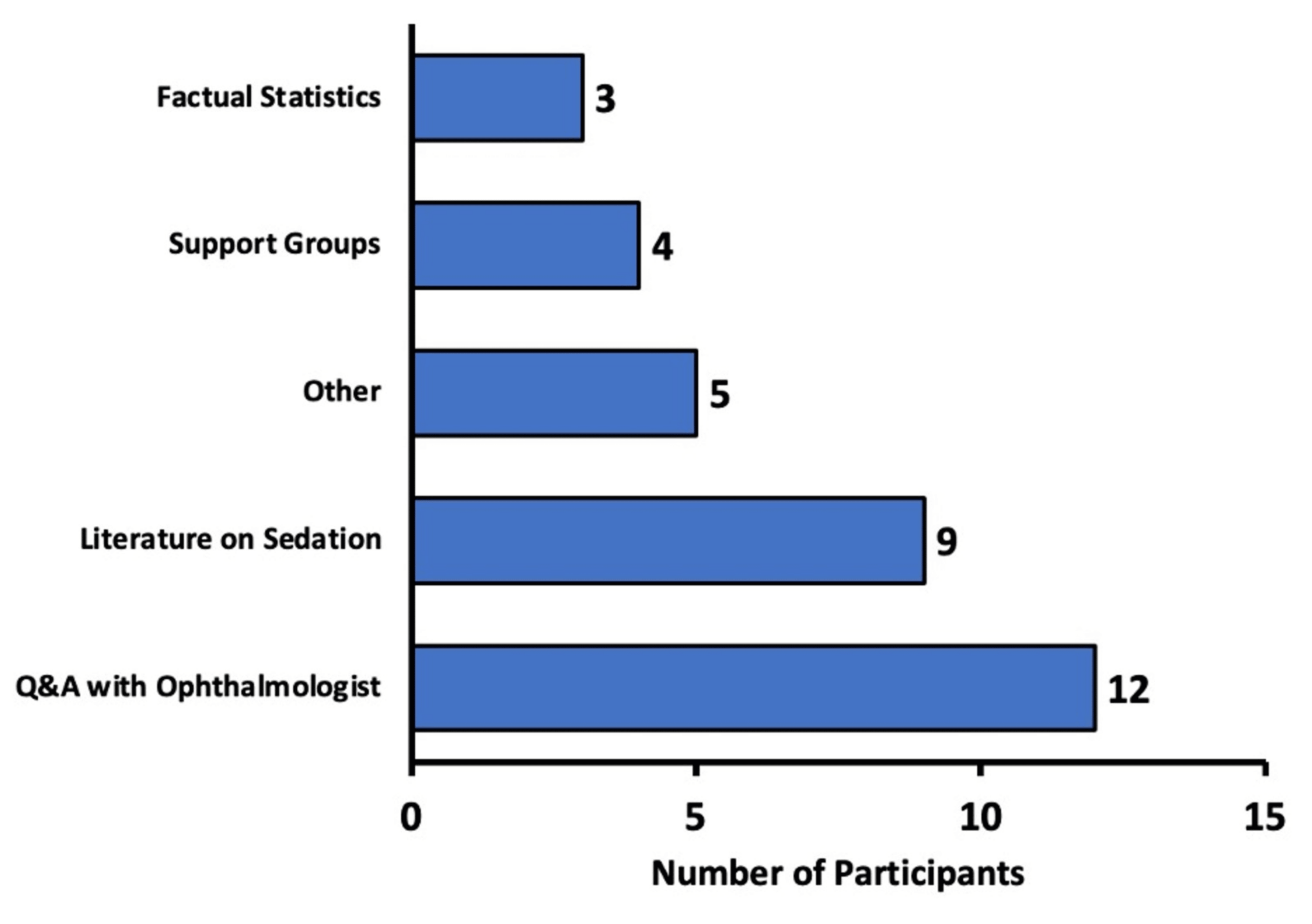
Recommended interventions by patients to improve the sedation experience Interventions that participants believed would ease their concerns about sedation. More than one option could be selected, so the total amount of responses (n=33) is greater than the number of participants (n=23).

Participants were asked about interventions they believed would most likely help them feel more comfortable about receiving sedation. Figure 5 demonstrates the various response options provided to participants. An “Other” option was also available for participants to provide their own ideas. Respondents could select multiple responses, so the total number of responses (n=33) was greater than the number of participants (n=23).

Of the respondents, 52.2% (n=12) believed a Q&A session with the ophthalmologist would help them feel more comfortable about receiving sedation. Thirty-nine point 1 percent (39.1%; n=9) suggested that receiving literature on sedation (e.g., pamphlets, flyers) would help. Seventeen point four percent (17.4%; n=4) recommended joining support groups with others who experienced sedation. Thirteen percent (13.0%; n=3) believed factual statistics demonstrating possible outcomes and adverse effects would address their concerns about sedation.

Among the “Other” responses, 8.7% (n=two) of participants suggested a therapy dog, and 8.7% (n=two) recommended a Q&A session with the anesthesiologist or nurse anesthetist. Four point three percent (4.3%; n=1) believed that music during the actual administration of sedation would help them feel more comfortable about the experience (Figure 5).

Self-reported side effects from sedation

Respondents were asked to self-report any side effects they may have experienced from sedation. Because this question was free response, and hence patients may list more than one side effect, the total number of responses (n=25) was greater than the number of participants (n=23).

**Figure 6:**
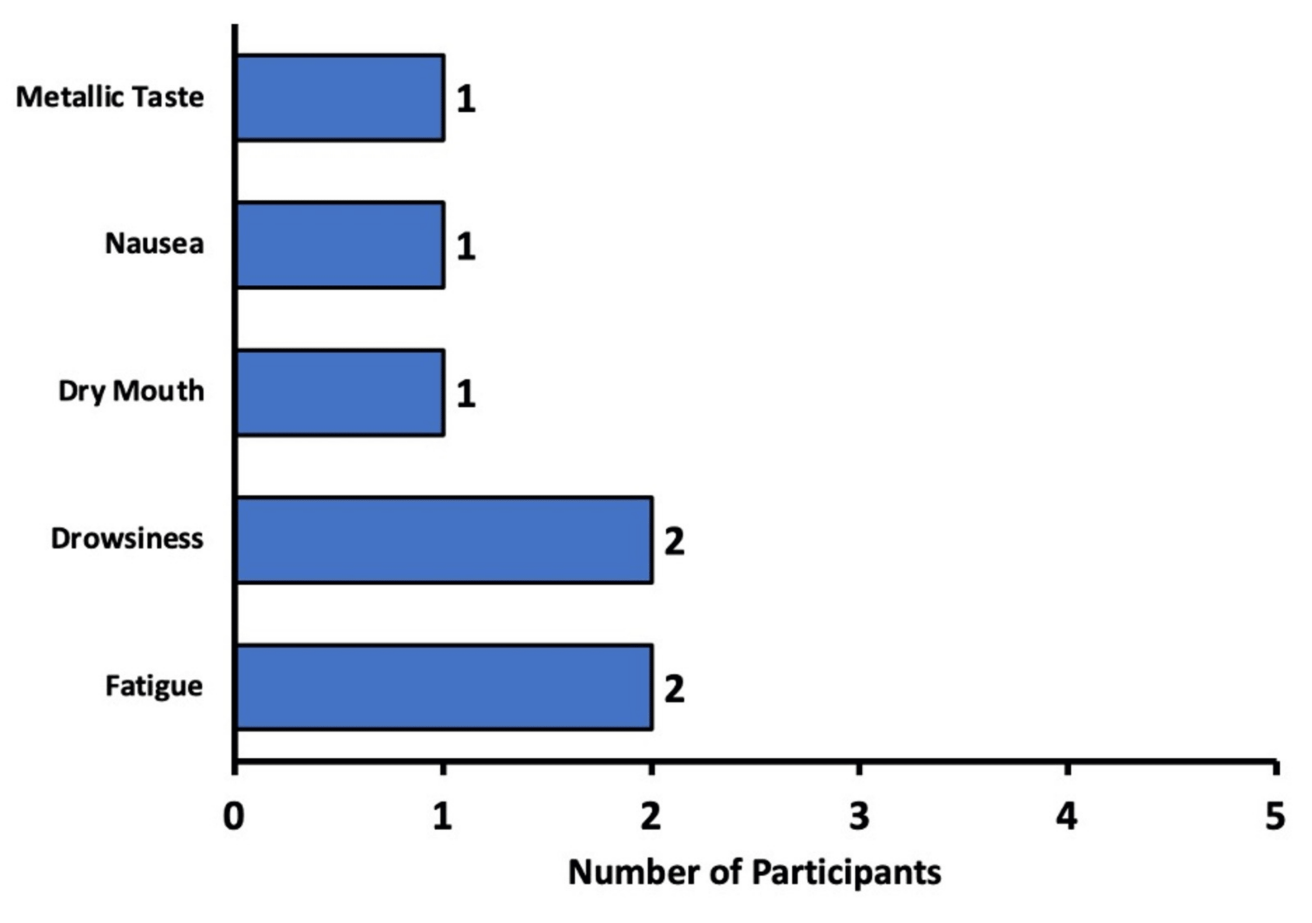
Patient-reported side effects from sedation Self-reported side effects from sedation in participants (n=seven). Participants who denied experiencing any side effects were excluded from the figure.

Of the participants, 78.3% (n=18) stated that they did not experience any side effects after sedation. In those who did self-report side effects, two experienced fatigue, two reported drowsiness, one experienced dry mouth, one developed nausea, and one reported a metallic taste in the mouth. Ultimately, no severe side effects from sedative administration were reported by any participants (Figure 6).

## Discussion

In our study, we analyzed the opinions of patients on sedation in ophthalmic procedures through a questionnaire study. Data were gathered on subjective experiences such as comparison of their anxiety levels before and after sedation, patient recommendations of sedatives to others, and self-reported potential side effects from sedation. Among the 23 survey responses, 21.7% considered canceling their recent procedure before receiving sedation, while 78.3% did not (Figure 1). After experiencing sedation for their procedure, 56.5% indicated that they would consider canceling future procedures if sedation were not offered (Figure 2). Notably, eight participants who initially responded “No” to the first question later responded “Yes” to the second. This suggests that while they may have initially been indifferent to sedation, their experience with the recent procedure positively changed their perspective, giving them the realization that they would not want to undergo a future procedure without a sedative.

These results are further supported by responses regarding the recommendation of sedation to others. Of the participants, 91.3% reported being either extremely likely or somewhat likely to recommend sedation to others. Only two participants (8.7%) were neutral, and no respondents were unlikely to recommend sedation to family or friends (Figure 4). These findings further highlight the patients’ satisfaction with sedation and their preference for its continued use in future procedures.

When comparing the perceived levels of anxiety before and after the eye procedures, 91.3% of our participants reported some level of anxiety before receiving sedation for their procedure. Our finding correlates with prior surveys that found preoperative anxiety to be prevalent, with studies reporting a wide range (11% to 89%) of surgical patients experiencing some anxiety regarding their procedure [8,9]. Common sources of anxiety include the outcome of the operation, the anesthesia process, and possible complications (e.g., vision loss) [8,10]. The average anxiety level in our respondents decreased from 4.61 to 1.65 after receiving sedation, reflecting a reduction of 2.96 (Figure 3). Levels of anxiety decreased for 78.3% of our participants and remained unchanged for 8.7% of our participants. These results align with the study by Venkatesh et al., which reported that midazolam significantly alleviated fear and discomfort in patients. In that study, 73.3% of patients who did not receive midazolam reported experiencing fear intraoperatively, compared to only 30.7% of those who did receive the sedative [3].

Interestingly, for 13% of our participants, levels of anxiety increased after receiving sedation for their procedure. The reason for this is unclear, but one possible theory is a rare paradoxical reaction to benzodiazepines, which occurs in less than 1% of patients. It is characterized by increased anxiety, agitation, hyperactivity, or talkativeness following benzodiazepine administration. Multiple case studies have documented this phenomenon [11-13], describing increased agitation following the administration of a benzodiazepine, and, in some cases, flumazenil administration decreased agitation. Further surveys and research may be indicated to better understand why some patients experienced heightened anxiety despite sedation.

In our study, we also aimed to explore interventions patients believed might alleviate their apprehensions about receiving sedation during ophthalmic surgery. The findings illustrate a clear preference for direct, personalized communication as the primary means of enhancing patient comfort. More than half of our respondents (52.2%) reported that a Q&A session with their ophthalmologist would be most reassuring. This preference underscores the central role that direct clinician-patient interactions play in addressing perioperative anxiety (Figure 5). Current literature suggests that most patients prefer face-to-face discussions with their surgeon (augmented by take-home brochures) over written pamphlets alone [8]. Direct personal communication allows patients to ask questions and clarify concerns in real time, building trust and understanding [8].

In addition to interactive communication, 39.1% of participants in our study supported the delivery of written literature such as pamphlets or flyers (Figure 5). Written materials provide patients with the opportunity to revisit complex information at their own pace, reinforcing verbal instructions and potentially aiding in the retention of important details regarding sedation risks and benefits. This dual approach to patient education - verbal and written - yields the best information retention and emotional comfort for patients [8].

Although a smaller proportion of our respondents indicated that joining support groups (17.4%) or receiving factual statistical data about outcomes and adverse effects (13.0%) would be beneficial, these strategies nonetheless have important adjunctive value (Figure 5). Peer support opportunities can offer real-life insights and reassurance from others who have experienced sedation. While direct evidence of support groups in ophthalmic surgery is limited, general elective surgery data indicate that leveraging social support systems can modestly reduce anxiety and help patients feel less lonely in the pre-surgery period [9].

A minority of respondents mentioned the potential benefit of a therapy dog (8.7%), music therapy (4.3%), and a Q&A session with the anesthesiologist or nurse anesthetist (8.7%). Although these responses represented a smaller segment of the overall feedback, they highlight the importance of a multidisciplinary approach, suggesting that comfort may also be enhanced by addressing the interpersonal and emotional facets of patient care (Figure 5).

With regard to side effects, most participants (78.3%) did not self-report any effects from sedation. For the nearly 22% of participants in our study who developed side effects, all were mild, and no severe side effects were experienced. Side effects in our respondents included a metallic taste, nausea, fatigue, and drowsiness (Figure 6). In the general population, the majority of side effects from sedatives are experienced by 1% or less of patients. The most common side effects experienced by patients undergoing sedation with benzodiazepines are hiccups, cough, nausea, and vomiting [14].

Although only one respondent (4%) in our survey experienced nausea, this does coincide with the reported side effect rate for nausea caused by midazolam, which is 2-5% [14]. Another notable side effect experienced by our respondents is a metallic taste. The side effects listed for a comparable sedative medication do not contain a metallic taste, but they do list an acidic taste that is experienced by less than 1% of those who use benzodiazepines [14]. These findings are commensurate with the overall effect of sedative medication. Side effects have a dose-response relationship that is dose-dependent, meaning that the higher the dose of the medication, the greater the probability of a side effect or severe side effect [15]. The fact that low doses are used for sedation in ophthalmic procedures may explain why most patients in our study did not experience any side effects [4,16].

Limitations

The sample size and the potential for recall bias are significant limitations of our study that must be addressed. The small sample size (n=23) may be explained by the six-month study period and the recruitment of participants solely from ophthalmologists in Alabama. Small sample sizes can introduce a higher risk of errors, reduce statistical power, and lower statistical significance [17,18]. In our study, the null hypothesis was rejected, as our p-value (0.0018) was under the alpha-value of 0.005, suggesting that our study had sufficient power despite the small sample size. However, this may also be explained by the magnitude of the association (standardized effect size = -0.738), rather than the inherent power of our study (80.4% by post hoc power analysis). Since the effect of sedation on reducing anxiety for ophthalmic procedures is strong, it can be detected even with a smaller sample size like ours. It is also important to acknowledge that while we rejected the null hypothesis, there is always a possibility of a Type I error [18]. Our study’s alpha-value was set to 0.005, indicating the very low likelihood of type I error. Nonetheless, the sample size - and hence the statistical power and significance - in our future questionnaires may be improved by lengthening the study period to a year or more and reaching out to ophthalmologists outside of Alabama to distribute surveys to a wider range of patients. Social media use for recruitment should also be limited, as online survey respondents may be disproportionately younger, resulting in a skewed sample.

Although we attempted to minimize the risk of recall bias by restricting the inclusion criteria to those having undergone an eye procedure within 12 months of the survey, the bias nonetheless remains a prominent issue. With any study that requests patients to recall information, inaccurate memories of experiences may significantly alter results. Furthermore, considering how sedation alters the mind, patient opinions before receiving sedation may be skewed from the patient’s actual preoperative perceptions. In our further studies, recall bias can be reduced by restricting the inclusion criteria to a smaller time frame (e.g., having undergone an eye procedure within the last 6 months instead of the last 12 months). Another potential solution is that the questionnaire may be split into two portions, in which the surveys about patient opinions before sedation are administered before the procedure, and then patients fill out the second survey after their ophthalmic procedure.

## Conclusions

The findings from the current survey indicate that sedation is successful in reducing preoperative anxiety in most patients. Most participants recommended sedation to family and friends and would consider canceling future procedures if sedation were not administered. Furthermore, a majority of patients did not experience side effects, and in the handful who did, only mild side effects from sedation were reported. Through this study, we have defined a need for future research with larger, more diverse samples (e.g., variations in age, ethnicity, and geographic location) to confirm our results and further explore the impact of sedation on patient anxiety and emotions in ophthalmic procedures.

## References

[1] Usmani B, Iftikhar M, Latif A, Shah SM (2019). Epidemiology of primary ophthalmic procedures performed in the United States. Can J Ophthalmol.

[2] Kumar CM, Seet E, Eke T, Irwin MG, Joshi GP (2019). Peri-operative considerations for sedation-analgesia during cataract surgery: a narrative review. Anaesthesia.

[3] Venkatesh R, Kenia H, Sengupta S, Gopalakrishna M, Au Eong KG (2021). Effect of intravenous sedation on patients' visual experience and vital signs during cataract surgery under topical anesthesia: a randomized controlled trial. Adv Ophthalmol Pract Res.

[4] Woo JH, Au Eong KG, Kumar CM (2009). Conscious sedation during ophthalmic surgery under local anesthesia. Minerva Anestesiol.

[5] Loots H, Wiseman R (2006). Agents for sedation in ophthalmic surgery: a review of the pharmacodynamics and clinical applications. Curr Anaesth Crit Care.

[6] Peeler CE, Villani CM, Fiorello MG, Lee HJ, Subramanian ML (2019). Patient satisfaction with oral versus intravenous sedation for cataract surgery: a randomized clinical trial. Ophthalmology.

[7] Sadlak N, Fiorello MG, Cabral HJ, Subramanian ML, Desai MA, Lee HJ (2022). Poor correlation of provider and patient satisfaction with anesthesia in ophthalmic surgeries: a secondary analysis of a clinical trial. Clin Ophthalmol.

[8] Obuchowska I, Konopinska J (2021). Fear and anxiety associated with cataract surgery under local anesthesia in adults: a systematic review. Psychol Res Behav Manag.

[9] Kok XL, Newton JT, Jones EM, Cunningham SJ (2023). Social support and pre-operative anxiety in patients undergoing elective surgical procedures: a systematic review and meta-analysis. J Health Psychol.

[10] Ramirez DA, Brodie FL, Rose-Nussbaumer J, Ramanathan S (2017). Anxiety in patients undergoing cataract surgery: a pre- and postoperative comparison. Clin Ophthalmol.

[11] Short TG, Forrest P, Galletly DC (1987). Paradoxical reactions to benzodiazepines--a genetically determined phenomenon?. Anaesth Intensive Care.

[12] Hall RC, Zisook S (1981). Paradoxical reactions to benzodiazepines. Br J Clin Pharmacol.

[13] Robin C, Trieger N (2002). Paradoxical reactions to benzodiazepines in intravenous sedation: a report of 2 cases and review of the literature. Anesth Prog.

[14] Lexidrug Lexidrug (2025). Midazolam: drug information. UpToDate.

[15] Porter RJ, Rogawski MA (2025). Antiseizure drugs. Basic & Clinical Pharmacology, 14e.

[16] Nordt SP, Clark RF (1997). Midazolam: a review of therapeutic uses and toxicity. J Emerg Med.

[17] Froud R, Rajendran D, Patel S (2017). The power of low back pain trials. A systematic review of power, sample size, and reporting of sample size calculations over time, in trials published between 1980 and 2012. Spine (Phila Pa 1976).

[18] Shreffler J, Huecker MR (2025). Type I and Type II Errors and Statistical Power. https://www.ncbi.nlm.nih.gov/books/NBK557530/.

